# Achalasia-induced catastrophe in pregnancy: Empyema, esophageopleural fistula, septic shock, fetomaternal survival, multidisciplinary management, and literature review. A case report

**DOI:** 10.1016/j.ijscr.2025.111863

**Published:** 2025-08-24

**Authors:** Ayto Addisu Negash, Zelalem Getahun Demissie, Henok Gashaw Asefa, Amanuel Dagabas Wakoya, Seid Aliye Abdulahi, Dawit Garedew Bacha

**Affiliations:** a Department of Emergency Medicine and Critical Care, St. Paul's Hospital Millennium Medical College, Addis Ababa, Ethiopia; b Department of Intensive Care Medicine, St. Paul's Hospital Millennium Medical College, Addis Ababa, Ethiopia; c Department of Emergency Medicine and Critical Care, Harmonia University, Oromia, Ethiopia

**Keywords:** Achalasia, Empyema, Esophageopleural fistula, Thoracotomy, Pregnancy, Fetomaternal survival

## Abstract

**Introduction and importance:**

Esophageal achalasia is a rare motility disorder characterized by impaired relaxation of the lower esophageal sphincter and absent peristalsis. Its occurrence during pregnancy is uncommon but can result in life-threatening complications. We present a clinically significant case highlighting the diagnostic and management challenges of achalasia in late pregnancy.

**Case presentation:**

A 30-year-old gravida 4, para 3 woman at 33 weeks' gestation presented with worsening cough, chest pain, vomiting, and signs of sepsis. Imaging showed right-sided empyema, cavitary lung lesions, and a markedly dilated esophagus consistent with achalasia. Chest tube drainage revealed purulent fluid mixed with gastric contents, raising suspicion of TEF and lastly EPF was confirmed via endoscopy and intraoperatively. She underwent right thoracotomy with decortication, esophageal perforation repair, and Heller myotomy. During ICU care, she delivered a healthy neonate vaginally. Follow-up CT at 10 days showed significant radiologic improvement and no ongoing fistula. The patient was discharged after one month with favorable maternal and neonatal outcomes.

**Clinical discussion:**

Achalasia-related complications such as esophageal perforation, tracheoesophageal(TEF), and EPF during pregnancy are extremely rare and pose diagnostic and management challenges. Pregnancy-related physiological changes can exacerbate achalasia symptoms and increase risk of severe outcomes. Timely surgical intervention combined with critical care and obstetric management is essential to optimize fetomaternal survival.

**Conclusion:**

This case highlights a rare and life-threatening complication of achalasia in pregnancy. Early diagnosis and a multidisciplinary approach are crucial for successful management and favorable fetomaternal outcomes.

## Highlights

• Esophageal achalasia during pregnancy, especially when complicated by Esophageopleural fistula (EPF), empyema thoracic, and septic shock, is an exceptionally rare clinical entity.
• The condition may mimic pulmonary disease, presenting primarily with respiratory symptoms rather than classic dysphagia, leading to significant diagnostic challenges.
• Timely, multidisciplinary intervention, including ICU care, thoracotomy, and Heller myotomy, was critical.
• Early diagnosis and prompt intervention led to successful outcomes, with survival of both mother and baby.

## 1 Introduction

### 1.1 Background

Esophageal achalasia is a rare esophageal motility disorder characterized by impaired peristalsis of the esophageal body and incomplete relaxation of the lower esophageal sphincter (LES) [1]. The condition can occur at any age but is most frequently diagnosed between the third and fifth decades of life, affecting males and females equally, with a prevalence of approximately 8 per million individuals [2]. Achalasia typically presents with dysphagia, regurgitation, vomiting, chest discomfort, and weight loss. In severe or neglected cases, complications such as esophageal dilation, aspiration pneumonia, or nutritional deficiencies may develop [3].

### 1.2 Rationale

Achalasia in pregnancy is rare due to its slow progression but can lead to serious complications, including maternal malnutrition, aspiration, preterm labor, fetal growth restriction, fetal demise, and maternal death [4]. The typical clinical presentation includes dysphagia, regurgitation, and vomiting; however, respiratory complications such as aspiration pneumonia may also occur in advanced cases [5]. Severe vomiting in achalasia can lead to life-threatening complications such as esophageal perforation and thoracic infections. This rare complication carries a high mortality rate, especially when associated with sepsis. Early treatment is crucial, as delays worsen outcomes. In pregnancy, overlapping gastrointestinal and respiratory symptoms make timely diagnosis difficult. This case illustrates a rare, severe presentation of late-pregnancy achalasia complicated by perforation, TEF, empyema, and septic shock, requiring urgent multidisciplinary care for maternal and fetal survival.

### 1.3 Guidelines & literature

This case report has been reported in line with the Updated and Revised 2025 SCARE guidelines [6].Few cases of achalasia with severe pregnancy complications have been reported, highlighting the need for high clinical suspicion. Treatment focuses on preventing contamination, controlling infection, and restoring gastrointestinal integrity [7]. Literature supports early surgical intervention in complicated cases [8], but successful management also requires broad-spectrum antibiotics, nutritional support, and appropriate obstetric care [9].

## 2 Case presentation

A 30 year old gravida 4, para 3 woman with a 9 month history of amenorrhea with uncertain last menstrual period presented to the emergency department with a two month history of progressively worsening productive cough and shortness of breath. These symptoms were accompanied by easy fatigability, pleuritic chest pain, intermittent low-grade fever, nausea, and frequent vomiting of ingested food. Her symptoms acutely worsened one week prior to presentation. The patient has no known medical, surgical, or psychiatric history. She denies alcohol or drug use, has no known allergies, and no family history of similar illness.

Upon arrival, physical examination revealed that the patient appeared acutely ill and in distress, with blood pressure 80/50 mmHg, heart rate 121 beats per minute, respiratory rate 30 breaths per minute, and So₂ 88 % on room air and temperature 38.4 °C. There was also decreased air entry in the lower two thirds of the right lung and mild epigastric tenderness. The fundal height was in line with a gestational age of 33 weeks.

**Fig. 1:**
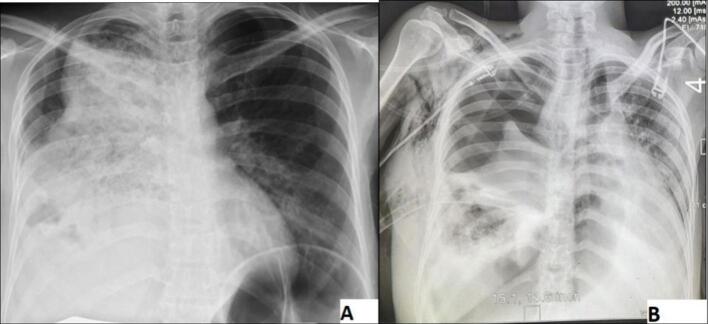
(A) An anteroposterior chest X-ray revealed right middle and lower zone opacification, obscured right heart border, and blunted cost phrenic angle and (B) a control chest x ray showing Chest tube insitu, partial lung re-expansion, and reduced pleural opacity.

Investigations revealed leukocytosis with neutrophilia, thrombocytosis, initial hyponatremia, and elevated BUN (Table 1). Bedside ultrasound reveal that there was large right side pleural collection with echo debris. She was initially managed with oxygen via a non-rebreather mask, fluid resuscitation, empirical intravenous antibiotics, and vasopressors, and was transferred to the intensive care unit (ICU) after she stayed for 6 h in the emergency department for management of septic shock of thoracic origin and impending respiratory failure. On ICU admission, the patient's clinical condition was worsening, with increasing respiratory distress, tachypnea, and oxygen desaturation despite receiving oxygen via a non-rebreather face mask. Bedside ultrasound revealed a significant right sided pleural collection. Following diagnostic thoracentesis, the pleural fluid tap revealed frank pus, confirming the presence of an empyema complicating aspiration pneumonia. In response, a right sided chest tube was inserted. Upon insertion, approximately 950 ml of thick, purulent fluid was immediately evacuated. Subsequent drainage over the next 24h yielded an additional 2 l of purulent fluid, indicating ongoing significant output from the pleural space. The initial chest X-ray demonstrated extensive airspace opacification in the right middle and lower lung zones, with visible air bronchograms, obscuration of the right heart border, and blunting of the right costophrenic angle. Following chest tube insertion, a control chest X-ray confirmed correct placement of the tube and demonstrated interval radiographic improvement, including partial re-expansion of the right lung, reduced effusion volume, and improved delineation of the right heart border and costophrenic angle. Despite ongoing drainage of purulent fluid, the observed radiographic changes suggest effective decompression of the pleural space and gradual resolution of compressive atelectasis (Fig. 1).

**Table 1:** Investigation summary.

Date	Oct 30	Nov12	Nov 16	Nov 19	Nov 21	Normal range
Complete Blood Count						
White Blood Cells	15.5	14.86	11.57	21.02	15.84	3.9–10.1 × 103 /mm3
Neutrophil	86	86.7	86.3	87.3	81.9	30.4–74.6 %
Lymphocyte	7.8	7.7	8.2	7.4	12.1	17.8–61.5 %
Hemoglobin	12.6	12.21	10.8	11.5	11	10.4–14.7 g/dl
Hemoglobin						34.4–48.3 %
Mean Corpuscular Volume	91.3	89.2	92.3	89.6	89.4	74.3–98.3 fl
Mean Corpuscular Hemoglobin	33.9	33.0	32.4	31.9	32.4	25.7–33.6 pg.
Platelets	317	329	551	475	532	100–300 × 103 /μL

Organ function test and electrolyte						
Blood Urea Nitrogen	37.1	–	–	25.6	–	16.6–48.5 mg/dl
Creatinine	0.14	–	–	0.35		0.7–1.3 mg/dl
Sodium	127	–	–			136–145 mmol/l
Potassium	3.35	–	–			3.5–5.5 mmol/l
ALT	–	–	–	4.3		0–40 IU
AST	–	–	–	12.3		0–45 IU

Coagulation profile						
PT	–	18.5	–	13.6		9.9–25 s
INR	–	1.3	–	1.2		
PTT	–	31.3	–	33.5		24–40 s

Pleural fluid analysis						
Total cell count	4930					cells/mm^3^
lymphocytes,	80					%
glucose	54					mg/dl
LDH	66					U/l
protein	0.4					g/dl
Gram stain,			Numerous gram-negative rods			
AFB smear			Negative			
Gene X-pert			negative			

AST Aspartate Aminotransferase, ALT Alanine Aminotransferase, LDH Lactate Dehydrogenase, INR International Normalized Ratio, PT Prothrombin Time, aPTT Activated Partial Thromboplastin Time, AFB Acid fast bacilli.

**Fig. 2:**
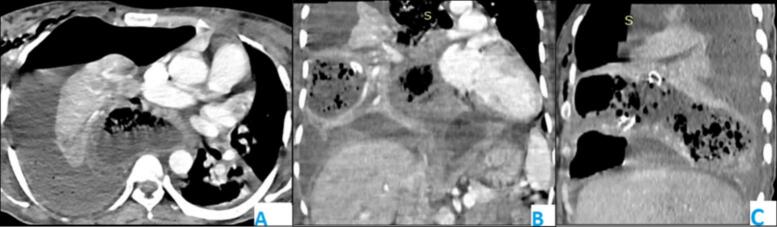
Thoracoabdominal Contrast-Enhanced CT Scan: (A) Axial, (B) Coronal, and (C) Sagittal views demonstrating a diffusely dilated esophagus consistent with achalasia, multifocal consolidation in the right lung, a cavitary lesion with an air-fluid level in the right lower lobe, and a moderate right pleural effusion with a chest tube in situ.

The day following ICU admission, the patient experienced a precipitous spontaneous vaginal delivery of a 2.1 kg male infant with APGAR scores of 7 and 8 at one and five minutes, respectively. Active management of the third stage of labor was performed, and the newborn was taken to the neonatal unit for further evaluation. On the second day post-chest tube insertion, persistent air leakage and the presence of gastric content in the drainage raised suspicion for TEF. A trial with an orange colored drink (Merinda) resulted in stained output in the chest drain, further supporting the suspicion. A contrast enhanced thoracoabdominal computed tomography (CT) scan was performed, revealing a diffusely dilated esophagus consistent with achalasia, multifocal consolidation in the right lung, a cavitary lesion with an air-fluid level in the right lower lobe, and a moderate right pleural effusion with a chest tube in place. These findings are indicative of aspiration pneumonia complicated by a lung abscess and Para pneumonic effusion. No evidence of TEF was identified on the chest CT (Fig. 2).

**Fig. 3:**
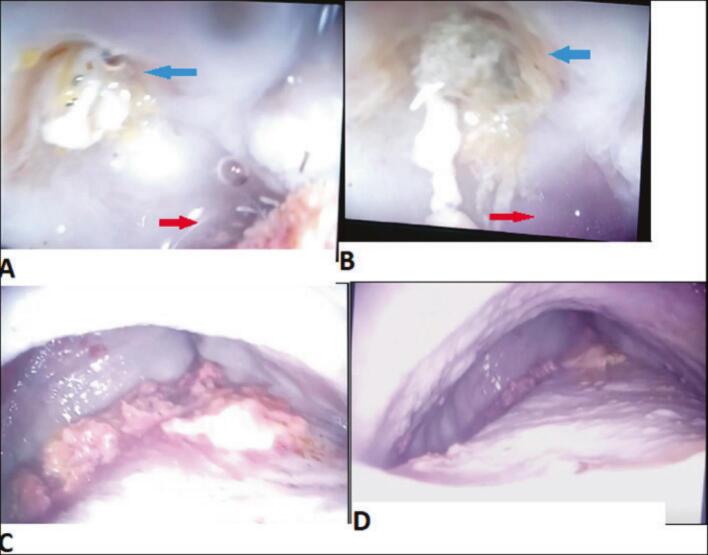
A & B: Endoscopic images showing a fistulous opening on the right esophageal wall (∼33 cm from the incisors) draining pus into the lumen (blue arrow), and a tightly stenosed lower esophageal sphincter (red arrow). C & D: Endoscopic views demonstrating a massively dilated, sigmoid-shaped esophagus with retained food debris. (For interpretation of the references to colour in this figure legend, the reader is referred to the web version of this article.)

On the third day, upper gastrointestinal endoscopy revealed a massively dilated, sigmoid shaped esophagus filled with food particles and a tightly stenosed lower esophageal sphincter. A fistulous opening was identified on the right esophageal wall approximately 33 cm from the incisors, with pus draining into the esophageal lumen up on inspiration. Findings were consistent with stage IV achalasia complicated by a esophageopleural Fistula (Fig. 3). A nasogastric tube was inserted endoscopically, and food particles retained in the dilated esophageal pouch were partially aspirated to reduce stasis and prevent further aspiration.

On the fourth day of ICU admission, the patient underwent a right posterolateral thoracotomy through the fifth intercostal space. Upon entering the pleural cavity, approximately 1500 ml of purulent fluid was drained. Intraoperative findings included extensive abscess formation involving the right lower lobe and diaphragmatic region, with areas of necrotic lung tissue and extension of the abscess into the lobar fissures. Careful dissection in the inflamed mediastinal field revealed a sealed perforation in the mid to distal esophagus. The fistula was directly visualized during dissection and identified based on localized inflammatory changes, soft fibrous adhesions, and proximity to the abscess cavity. Although not evident on imaging, the preoperative endoscopy helped localize the area of interest and guided the surgical team's focus; intraoperative endoscopy was not performed. A thoracotomy with decortication and drainage of the lung abscess was performed, followed by reinforcement of the sealed esophageal perforation. After completing the thoracic portion, an upper abdominal midline incision was made to access the lower esophagus and perform a 10 cm Heller myotomy, with preservation of the anterior vagus nerve. A nasogastric tube was inserted under direct vision into the stomach, and all surgical incisions were closed in layers. The procedure was complicated by intraoperative hypotension requiring vasopressor support and transfusion of one unit of blood.

Pleural fluid analysis showed 4930 cells/mm^3^ with 80 % lymphocytes, glucose 54 mg/dl, LDH 66 U/l, and protein 0.4 g/dl. Gram stain revealed numerous gram negative rods, while AFB smear and Gene xpert MTB testing were negative.

Postoperatively, the patient was kept nil per os, maintained on broad spectrum antibiotics (meropenem and vancomycin), and managed supportively in the ICU. She remained hemodynamically stable, with resolution of sepsis and no further evidence of esophageal leakage.

**Fig. 4:**
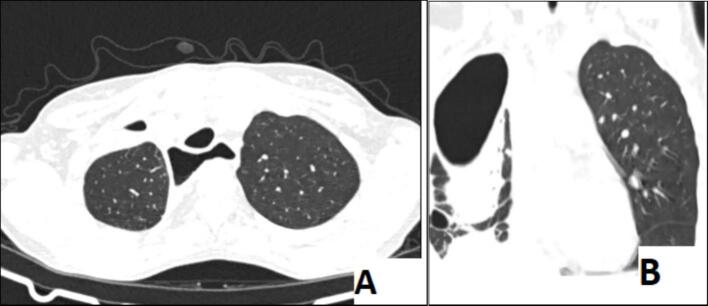
A (Axial), B (Sagittal), and C (Coronal) follow-up thoraco abdominal contrast-enhanced CT scan demonstrated decreased right pleural effusion, reduced size of the right lower lobe cavitary lesion with surrounding consolidation, and resolution of multifocal right lung opacities. The esophagus remains dilated with reduced intraluminal stasis.

On the 10th day of the ICU admission, the patient showed significant improvement in her clinical condition and, follow up contrast enhanced CT of the thorax demonstrated notable radiologic improvement evidenced by the right pleural effusion had significantly decreased. The right lower lobe cavitary lesion showed a reduction in size and wall thickness, with decreased surrounding consolidation. Multifocal right lung opacities had largely resolved. The esophagus remained dilated but showed decreased intraluminal stasis, suggesting improved esophageal emptying. No new complications were identified, and there remained no evidence of a tracheoesophageal fistula (Fig. 4).

Following thoracotomy, the chest tube initially drained purulent fluid and air, which gradually decreased over the first 10 postoperative days. By day 10, the drainage had resolved completely, with no further pus or air leak observed. The chest tube was removed on the 12th postoperative day. The patient was subsequently transferred to the surgical ward on the 20th day of ICU admission. The patient was discharged after one month of hospitalization with significant clinical improvement. Both maternal and neonatal outcomes were favorable, with survival of both.

## 3 Discussion

Achalasia during pregnancy is a rare condition characterized by incomplete relaxation of the lower esophageal sphincter and impaired esophageal peristalsis [10]. As of 2021 there was fewer than 40 reported cases in the literature [11]. This case of a 30-year-old gravida 4 para 3 female presenting with empyema thoracic and septic shock highlights several unique clinical, diagnostic, and therapeutic challenges that require detailed discussion.

The common symptoms of esophageal achalasia are dysphagia, chest pain, regurgitation, and weight loss. The patient's presentation of productive cough, pleuritic chest pain, and septic shock underscores the severe respiratory and systemic implications of achalasia in its advanced stages. Pregnancy further complicates the clinical picture, as overlapping symptoms like nausea, vomiting, and fatigue may obscure the diagnosis making it more challenging [11]. Pregnancy can exacerbate underlying conditions like achalasia [12]. Moreover, physiological changes during pregnancy, such as increased intra-abdominal pressure and hormonal influences on smooth muscle, may worsen the esophageal dysfunction, increasing the risk of complications [13].

Differentiating empyema secondary to esophageal perforation from other causes, such as primary esophageal rupture (Boerhaave syndrome), is critical but challenging. The drainage of gastric contents via the chest tube raised suspicion for esophageal rupture [14]. Our clinical suspicion is further confirmed by chest CT imaging and upper GI endoscopy.

Esophageal perforation is a rare but serious complication of achalasia, as reported in previous studies [15,16]. It can lead to severe outcomes, including aspiration pneumonia, septic shock, and fetal growth restriction [10,17]. EPF is an uncommon condition, despite of an anatomical proximity of these structures [18]. Affected individuals may present with signs and symptoms of empyema, such as chest pain, high fever, and hypotension. The presence of food particles or gastric contents in the pleural space highly suggests this diagnosis [19].

In the context of pregnancy, the management of esophageal achalasia becomes even more complex, particularly when complications such as empyema thoracic and septic shock arise. Septic shock during pregnancy, though rare, poses a significant risk to both maternal and fetal health, with maternal mortality rates ranging from 20 % to 28 % in affected individuals [20]. The management of septic shock in pregnant patients requires immediate recognition of the source of infection and targeted therapy [20,21]. Management strategies also vary, with conservative approaches often preferred during pregnancy. These may include total parenteral nutrition [3] or bridging therapies until after delivery [11]. Definitive treatments, such as laparoscopic esophageal myotomy or per oral endoscopic myotomy, are typically performed postpartum [17,3].

Our patient was initially treated with broad-spectrum antibiotics and chest tube drainage. Following delivery, she developed ongoing high-output chest tube drainage containing pus and ingested material. Endoscopic evaluation confirmed an esophageal perforation with an esophageopleural fistula, evidenced by bidirectional movement of pleural contents during respiration, shows persistent contamination and systemic infection [22].

Given the severity of pleural contamination and signs of sepsis, conservative management was deemed insufficient. Large or uncontained esophageal perforations with ongoing mediastinal or pleural infection generally require prompt surgical intervention, as non-operative approaches are associated with higher morbidity and mortality [23].

The patient underwent a right posterolateral thoracotomy, which allowed for effective decortication and primary repair of the esophageal perforation with reinforcement—essential steps to achieve optimal source control in thoracic esophageal leaks [23]. A concurrent esophageal myotomy was performed to address underlying achalasia, a condition that predisposes to perforation through chronic esophageal distension and increased intraluminal pressure [24].

Achalasia during pregnancy presents unique management challenges, often necessitating individualized treatment strategies that balance maternal and fetal safety. While less invasive options, such as botulinum toxin injection or pneumatic dilation, may serve as temporary measures, definitive therapy such as surgical or endoscopic myotomy becomes essential when life-threatening complications occur [11]. Both laparoscopic Heller myotomy and POEM have demonstrated high efficacy in non-pregnant populations, and emerging case reports support their safe use during pregnancy when clinically indicated [22].

In this case, early surgical intervention addressed both the immediate threat of sepsis and the underlying motility disorder, aligning with standard surgical principles that prioritize timely source control and definitive repair in complex thoracic and esophageal pathologies [23].

In summary, esophageal achalasia can lead to significant respiratory complications, particularly in pregnant patients, where the risk of septic shock and empyema thoracic can further complicate management. Early identification and intervention are crucial to improve outcomes for both the mother and the fetus, highlighting the need for a comprehensive understanding of the interactions between these conditions.

## 4 Conclusion

This case highlights an exceptionally rare and life threatening presentation of esophageal achalasia in late pregnancy, complicated by spontaneous esophageal perforation, tracheoesophageal fistula, empyema thoracic, and septic shock. The clinical course underscores the diagnostic and therapeutic challenges posed by overlapping gastrointestinal and respiratory symptoms during pregnancy, which can obscure timely recognition of serious underlying conditions.

Despite the complexity, a multidisciplinary approach, combining intensive care support, timely surgical intervention (thoracotomy, decortication, esophageal repair, and Heller myotomy), and obstetric management, resulted in a remarkable outcome, with both maternal recovery and the delivery of a viable neonate. This case reinforces the importance of early suspicion, aggressive source control, and individualized, coordinated care in managing rare thoracoabdominal emergencies during pregnancy.

## Consent for publication

Written informed consent was obtained from the patient for publication of this case report and use of images. A copy of the written consent is available for review by the Editor-in-Chief of this journal upon request.

## Ethical approval

Ethical approval is deemed unnecessary by St. Paul's Hospital Millennium Medical College Institutional Review Board, as this is a rare case faced during clinical practice and doesn't involve experiments in humans or animals.

## Funding

There is no source of funding for this manuscript.

## Author contribution

**Ayto Addisu Negash:** Study conceptualization and design, original draft write-up, data curation, paper review & editing, and patient management.

**Zelalem Getahun Demisie:** Study conceptualization and design, original draft write-up, data curation, paper review & editing, and patient management.

**Henok Gashaw Asefa:** Study conceptualization and design, original draft write-up, data curation, paper review & editing, and patient management.

**Amanuel Dagabas Wakoya:** Study conceptualization and design, original draft write-up, data curation, paper review & editing, and patient management.

**Seid Aliye Abdulahi:** Study conceptualization and design, original draft write-up, data curation, paper review & editing, and patient management.

**Dawit Garedew Bacha:** Study conceptualization and design, original draft write-up, data curation, paper review & editing, and patient management.

## Guarantor

Ayto Addisu Negash.

## Research registration number

Not applicable.

## Disclosure

The case has not been presented at any conferences or regional meetings, nor is it currently under review by any other journal. This ensures its originality and the opportunity for fresh insights within the academic community.

## Conflict of interest statement

The authors declare that they have no conflict of interest.
